# Health Care Service Utilization among Patients with Bladder Pain Syndrome/Interstitial Cystitis in a Single Payer Healthcare System

**DOI:** 10.1371/journal.pone.0087522

**Published:** 2014-01-29

**Authors:** Shiu-Dong Chung, Shih-Ping Liu, Hsien-Chang Li, Herng-Ching Lin

**Affiliations:** 1 Division of Urology, Department of Surgery, Far Eastern Memorial Hospital, Ban Ciao, Taipei, Taiwan; 2 Sleep Research Center, Taipei Medical University Hospital, Taipei, Taiwan; 3 Department of Urology, National Taiwan University Hospital and College of Medicine, Taipei, Taiwan; 4 School of Health Care Administration, Taipei Medical University, Taipei, Taiwan; Oklahoma University Health Sciences Center, United States of America

## Abstract

**Background:**

This study aims to investigate the differences in the utilization of healthcare services between patients with bladder pain syndrome/interstitial cystitis (BPS/IC) and patients without using a population-based database in Taiwan.

**Methods:**

This study comprised of 350 patients with BPS/IC and 1,750 age-matched controls. Healthcare resource utilization was evaluated in the one-year follow-up period as follows: number of outpatient visits and inpatient days, and the mean costs of outpatient and inpatient treatment. A multivariate regression analysis was used to evaluate the relationship between BPS/IC and total costs of health care services.

**Results:**

For urological services, patients with BPS/IC had a significantly higher number of outpatient visits (2.5 vs. 0.2, *p*<0.001) as well as significantly higher outpatient costs ($US166 vs. $US6.8, *p*<0.001) than the controls. For non–urologic services, patients with BPS/IC had a significantly high number of outpatient visits (35.0 vs. 21.3, *p*<0.001) as well as significantly higher outpatient cots ($US912 vs. $US675, *p*<0.001) as compared to the controls. Overall, patients with BPS/IC had 174% more outpatient visits and 150% higher total costs than the controls. Multiple-regression-analyses also showed that the patients with BPS/IC had significantly higher total costs for all healthcare services than the controls.

**Conclusions:**

This study found that patients with BPS/IC have a significantly higher number of healthcare related visits, and have significantly higher healthcare related costs than age-matched controls. The high level of healthcare services utilization accrued with BPS/IC was not necessarily exclusive for BPS/IC, but may have also been associated with medical co-morbidities.

## Introduction

Interstitial cystitis (IC) is a chronic condition characterized by increased urinary frequency, urgency, nocturia, dyspareunia and/or pelvic pain not associated with any known etiology [1], [2]. The prevalence of IC is estimated to range between 0.45% and 12.6% worldwide [3], [4]. One study even suggested that IC may affect as many as 20% of women [5]. Patients with IC may not only have a reduced quality of life, but are also more likely to suffer from other physical or mental co-morbidities as compared to the general population [6], [7]. Furthermore, studies have reported that IC has created substantial medical costs through the increased volume of healthcare service utilization [8].

IC is a costly disease to the health care system. Clemens et al. reported that the mean yearly costs were 2.4-fold greater for the patients with IC than for controls without in a managed care population [9]. Another study by the same research group showed that patients with IC have 2- to 3-fold higher direct costs as compared to patients without IC [10]. Wu et al. indicated that the average IC patient has 130% higher direct costs than the average non-IC patient based on claims data from several managed care plans [11]. One study by Payne et al. also found that a diagnosis of IC was associated with a 2-fold increase in direct medical costs in the US [12]. However, all the studies on the costs behind IC have relied on samples taken from subpopulation in the US (eg, those covered by a specific insurance plan). To our knowledge, no such studies have been conducted in other regions or countries. The study of the economic burden on the healthcare system attributable to IC has been lacking in Asian countries.

The aim of this study is to investigate the differences in the utilization of healthcare services between patient cases with BPS/IC and aged-matched controls without the condition using Taiwan's National Health Insurance (NHI) population-based database. Taiwan initiated a single-payor NHI program in 1995. Over 99% of the 23million citizens are covered by the NHI, providing a comprehensive benefit coverage with very low co-payments (about $US5 per outpatient visit) regardless of socio-economic status. Therefore, unlike many of the healthcare plans in the US which place restrictions on the access to medical care, the people of Taiwan have free access and an open choice to providers throughout the country. This provides researchers a better opportunity to explore the utilization of healthcare services for patients with BPS/IC.

## Methods

### Database

Data on the sampled subjects and their utilization of healthcare services were retrieved from the “Longitudinal Health Insurance Database (LHID2000)”. The LHID2000 contains complete medical claims and registration files for 1,000,000 enrollees, who are randomly selected from all enrollees listed in the 2000 Registry of Beneficiaries (*n* = 23.72 million) under the National Health Insurance (NHI) program. Taiwan has been implementing the NHI program since 1995. The Taiwan National Health Research Institute and other researchers have validated the accurate representation of the LHID2000 relative to the entire population of NHI enrollees in terms of gender distribution. In addition, hundreds of studies have been published using the data from the NHI program.

This study was exempt from a full review by the Institutional Review Board of Taipei Medical University because the LHID2000 consists of de-identified secondary data released to the public for research purposes.

### Study Sample

This cross-sectional study first identified 512 female patients who had received a first-time diagnosis of BPS/IC (ICD-9-CM code 595.1 (chronic IC)) during an ambulatory care visit (including outpatient departments of hospitals and clinics) between January 1, 2006 and December 31, 2010. Although BPS/IC patients can be prescribed different medications in other regions or countries, in accordance with the standardized procedures of the NHI program, coverage for the medication Cystistat® is exclusively reserved for patients diagnosed with BPS/IC in Taiwan. Due to the stringent oversight of peer reviews for the prescription of Cystistat®, it is unlikely that misdiagnosed cases were included in our study group. Therefore, in order to increase the validity of the BPS/IC diagnoses, this study only included BPS/IC patients who had received a prescription for Cystistat® (sodium hyaluronate) (*n* = 361). We excluded patients <18 or >80 (*n* = 11) years of age. The date of the first ambulatory care visit in which the patient received their BPS/IC diagnosis was recorded as the index date. Overall, there were 350 BPS/IC patient cases in the study group.

For the controls, we also retrieved the data from the LHID2000. We identified female subjects aged between 18 and 79 years old. Subjects who already had a prior history of BPS/IC were excluded from the study. We then randomly selected 1,750 controls (5 controls per patient BPS/IC case) matched to the study cases by age (18∼39, 40∼49, 50∼59, 60∼69, and 70∼79 years) and index year through a SAS proc surveyselect program. The year of the index date for the controls was simply a matched year in which the controls visited a physician. We likewise indicated the date of their first visit of a physician occurring during that matched year as the index date for controls. As a result, there were 2100 study subjects including 350 cases with BPS/IC and 1750 controls in this study.

### Variables of interest

The utilization of healthcare services was evaluated in the one-year follow-up period following the index date and was defined as mean±SE per group of the following: number of outpatient visits and inpatient days and the mean costs of outpatient and inpatient treatments. The utilization of healthcare services was also separated into urological and non-urological services. We defined urological service as service provided by a certified urologist.

### Statistical Analysis

We used a SAS statistical package (SAS System for Windows, vers. 8.2, Cary NC, USA) to conduct the statistical analyses. Descriptive statistical analyses, including frequency, percentage, mean and standard deviation, were performed on all of the outcome variables. Student's t-tests were carried out in order to examine the relationships between the outcome variables for patients with BPS/IC and controls. Furthermore, a multivariate-regression-analysis was used to model the logarithm of mean costs as a linear function of a set of independent variables. The difference was considered significant if a two-sided *p* value was ≤0.05.

## Results

Of the 2,100 female study subjects, the mean age was 48.4±13.8 years old; and about 50% were older than 50 (Table 1). After matching for age and the year of the index date, patients with BPS/IC were found to reside, more likely than controls, in the most urbanized areas and have monthly incomes ≤NT$15,841. However, there was no significant difference in the geographic region between patients with BPS/IC and controls (*p* = 0.159). A summary of the use and costs of healthcare services within the one-year period following the index date for patients with BPS/IC and controls is provided in Table 2.

**Table 1:** Demographic characteristics of subjects with bladder pain syndrome/interstitial cystitis and comparison subjects (n = 2100).

Variable	Subjects with bladder pain syndrome/interstitial cystitis (*n* = 350)		Comparison subjects (*n* = 1750)		*p* value
	Total no.	Percent (%)	Total no.	Percent (%)	
Age (years)					>0.999
<40	84	24.0	420	24.0	
40∼49	90	25.7	450	25.7	
50∼59	87	24.9	435	24.9	
60∼69	58	16.6	290	16.6	
70∼79	31	8.9	155	8.9	
Urbanization level					0.008
1 (most urbanized)	140	40.0	566	32.3	
2	89	25.4	512	29.3	
3	40	11.4	266	15.2	
4	56	16.0	230	13.1	
5 (least urbanized)	25	7.1	176	10.1	
Monthly income					0.007
NT$1∼15,840	159	45.4	675	38.6	
NT$15,841∼25,000	137	39.1	686	39.2	
≥NT$25,001	54	15.4	389	22.2	
Geographic region					0.159
Northern	196	56.0	868	49.6	
Central	73	20.9	398	22.7	
Southern	76	21.7	448	25.6	
Eastern	5	1.4	36	2.1	

**Table 2:** Use and costs of health care services within one year for subjects with bladder pain syndrome/interstitial cystitis and comparison subjects.

Variable	Subjects with bladder pain syndrome/interstitial cystitis (*n* = 350)		Comparison subjects (*n* = 1750)		*p* value
	Mean	SE	Mean	SE	
Urologic services					
Outpatients services	2.5	4.7	0.2	1.1	<0.001
Outpatient costs	166	449	6.8	58.7	<0.001
Inpatient days	–	–	–	–	–
Inpatient costs	–	–	–	–	–
Total costs	166	449	6.8	58.7	<0.001
Non-urologic services					
Outpatients services	35.0	26.0	21.3	17.1	<0.001
Outpatient costs	912	1235	675	1852	<0.001
Inpatient days	1.22	5.83	0.88	5.69	0.305
Inpatient costs	290	124	230	157	0.727
Total costs	1149	2069	900	2519	<0.001
All health services					
Outpatients services	37.4	26.8	21.5	17.3	<0.001
Outpatient costs	1078	1372	682	1854	<0.001
Inpatient days	1.22	5.83	0.88	5.69	0.305
Inpatient costs	290	124	230	157	0.727
Total costs	1367	2310	912	2526	0.002

On the utilization of urological services patients with BPS/IC had significantly more outpatient visits (2.5 vs. 0.2, *p*<0.001) as well as significantly higher outpatient costs ($US166 vs. $US6.8, *p*<0.001) as compared to controls. As a whole, 257 of the 2,100 sampled subjects (12.2%) had ever used urological services during the one-year follow-up period— 45.4% of patients with BPS/IC and 5.6% of controls.

There were no inpatient days or costs for urological services in either group. In other words, the mean number of yearly outpatient visits and the mean yearly costs for urological services within the follow-up period were 12.5-fold and 24.4-fold greater, respectively, for patients with BPS/IC than controls.

As for the utilization and costs of non–urological services, patients with BPS/IC had significantly more outpatient visits (35.0 vs. 21.3, *p*<0.001) as well as a significantly higher outpatient ($US912 vs. $US675, *p*<0.001) and total ($US1149 vs. $US900, *p*<0.001) costs than controls. There was no significant difference in the number of inpatient days and inpatient costs between patients with BPS/IC and controls.

Table 2 also shows that for the use and costs of all healthcare services, patients with BPS/IC had significantly more outpatient visits (37.4 vs. 21.5, *p*<0.001) and significantly higher outpatient ($US1078 vs. $US682, *p*<0.001) and total ($US1367 vs. $US912, *p*<0.001) costs than controls. The average patient with BPS/IC had 174% more outpatient visits and 150% higher total costs than the average control.

The adjusted relationship between the total costs for all healthcare services and presence of BPS/IC is provided in Table 3. Multiple-regression-analyses showed that after adjusting for the factors of urbanization level, monthly income, and geographic region, patients with BPS/IC had significantly higher total costs for all healthcare services than controls.

**Table 3:** Multiple regression analysis for adjusted relationships between log costs of all health services and bladder pain syndrome/interstitial cystitis.

Variable	Log (all health services costs)	
	B	*p* value
Group		
Subjects with BPS/IC	0.780	<0.001
Controls		
Urbanization level		
1 (most urbanized)		
2	0.013	0.846
3	−0.157	0.069
4	0.033	0.721
5 (least urbanized)	0.005	0.962
Monthly income		
NT$1∼15,840		
NT$15,841∼25,000	−0.093	0.060
≥NT$25,001	−0.163	0.072
Geographic region		
Northern		
Central	0.016	0.074
Southern	0.056	0.068
Eastern	0.221	0.196

## Discussion

Little is known about the direct healthcare costs accrued by BPS/IC outside of the US. This is the first population-based study to compare the difference in the utilization of healthcare services between patients with BPS/IC and aged-matched controls without BPS/IC in an Asian country. We found that for the use of urological services, female patients with BPS/IC had significantly more outpatient visits (2.5 vs. 0.2, *p*<0.001) and higher outpatient costs ($US166 vs. $US6.8, *p*<0.001) than controls. Furthermore, for healthcare services as a whole, the total costs were 1.5-fold ($US1367 vs. $US912) greater for patients with BPS/IC than for controls. Even after adjusting for potential confounding factors, female patients with BPS/IC still had significantly higher total costs for all healthcare services than controls. The study demonstrates that patients diagnosed with BPS/IC consume a significantly higher amount healthcare resources than those without BPS/IC in Taiwan.

The results of these analyses are remarkably consistent with those in the US although the magnitude in cost differences between patients with BPS/IC and aged-matched controls is slightly different. In the US, previous studies have reported that the mean costs were ranged from 1.3- to 3-fold greater for the patients with IC than for the controls [9]–[12]. Our study found that the total costs were 1.5-fold greater for the patients with BPS/IC than for controls in Taiwan. The similarity in the results may imply that the findings can be broadly generalizable to populations at large.

As expected, the present study found that female patients with BPS/IC had significantly more outpatient visits for urological services than controls (mean value 2.5 vs. 0.2) during the follow-up period. Of the 350 female patients with BPS/IC, 159 (45.4%) had ever used an outpatient visit during the follow-up period. In Taiwan, it is very possible that many female patients with BPS/IC are seen by physicians specializing in obstetrics/gynecology. More than 80% of these urological outpatient visits were associated with BPS/IC. However, only 5.6% of controls had ever utilized a urological outpatient visit during the one-year follow-up period. The majority of these visits received the principal diagnoses of either urinary tract infection, hematuria, or calculus of ureter.

Our study also revealed that female patients with BPS/IC had significantly more outpatient visits (35.0 vs. 21.3) for non-urological reasons as well as significantly higher total costs ($US1149 vs. $US900) than controls. The excess costs may be due to the high prevalence of medical co-morbidities associated with BPS/IC. Although not investigated in the current study, one prior study by Keller et al. identified 9,269 subjects with BPS/IC and randomly matched 46,345 subjects without BPS/IC in Taiwan [7]. They suggested that patients with BPS/IC were more likely to have their medical co-morbidities (31 conditions studied) investigated than those without BPS/IC. Future studies are encouraged to investigate whether more accurate and earlier treatment could reduce healthcare costs related to medical co-morbidities.

The principal strength of our study was in the use of a population-based dataset. This mitigates the effect of selection bias inherent in voluntary registries and hospital-referred study patients. In addition, the database used in this study enabled us to trace the healthcare utilization of all study subjects, and this can reduce the potential recall bias present in a survey study.

Although these results provide large-scale epidemiologic data to the existing studies regarding the costs associated with BPS/IC, they must be seen in the light of a number of limitations. First, the BPS/IC diagnoses relied on administrative claims data reported by physicians and hospitals. The specific code of BPS/IC may have been overused by urologists. However, this study only included BPS/IC patients who had received a prescription for Cystistat® to increase the validity of the diagnosis. In addition, this standardized BPS/IC treatment procedure can reduce the confounding effects of treatment modalities on the costs. Also, this study only provides a cross-sectional analysis of the costs incurred by patients with BPS/IC. Although we only included newly diagnosed BPS/IC cases, this study did not compare the difference in the costs before and after the BPS/IC diagnosis. The high level of use and costs of healthcare services accrued by BPS/IC were not necessarily exclusive for BPS/IC but may have been associated with medical co-morbidities. Finally, this study did not evaluate indirect costs associated with BPS/IC. To our knowledge there is no available data about indirect costs in Taiwan.

Despite these limitations, this study found that female patients with BPS/IC had significantly higher use and costs of all healthcare services than aged-matched controls.
